# Distribution of calcium-binding proteins immunoreactivity in the bottlenose dolphin entorhinal cortex

**DOI:** 10.3389/fnana.2024.1321025

**Published:** 2024-02-05

**Authors:** Jean-Marie Graïc, Annamaria Grandis, Simona Sacchini, Claudio Tagliavia, Giulia Salamanca, Bruno Cozzi, Cristiano Bombardi

**Affiliations:** ^1^Department of Comparative Biomedicine and Food Science, University of Padova, Legnaro, Italy; ^2^Department of Veterinary Medical Sciences, University of Bologna, Bologna, Italy; ^3^Department of Morphology, University of Las Palmas de Gran Canaria, Las Palmas de Gran Canaria, Spain; ^4^Department of Veterinary Medicine, University of Teramo, Teramo, Italy

**Keywords:** entorhinal cortex, calretinin, calbindin-D28k, parvalbumin, bottlenose dolphin

## Abstract

**Introduction:**

The entorhinal cortex has been shown to be involved in high-level cognitive functions in terrestrial mammals. It can be divided into two main areas: the lateral entorhinal area (LEA) and the medial entorhinal area (MEA). Understanding of its structural organization in cetaceans is particularly important given the extensive evidence for their cognitive abilities. The present study describes the cytoarchitectural and immunohistochemical properties of the entorhinal cortex of the bottlenose dolphin (*Tursiops truncatus*, Montagu, 1821), perhaps the most studied cetacean species and a paradigm for dolphins and other small cetaceans.

**Methods:**

Four bottlenose dolphins’ entorhinal cortices were processed. To obtain a precise overview of the organization of the entorhinal cortex we used thionin staining to study its laminar and regional organization, and immunoperoxidase technique to investigate the immunohistochemical distribution of three most commonly used calcium-binding proteins (CBPs), calbindin D-28k (CB), calretinin (CR) and parvalbumin (PV). Entorhinal cortex layers thickness were measured, morphological and morphometric analysis for each layer were conducted and statistically compared.

**Results:**

Six layers in both the LEA and MEA were identified. The main difference between the LEA and the MEA is observed in layers II and III: the neurons in layer II of the LEA were denser and larger than the neurons in layer II of MEA. In addition, a relatively cell-free zone between layers II and III in LEA, but not in MEA, was observed. The immunohistochemical distribution of the three CBPs, CB, CR and PV were distinct in each layer. The immunostaining pattern of CR, on one side, and CB/PV, on the other side, appeared to be distributed in a complementary manner. PV and CB immunostaining was particularly evident in layers II and III, whereas CR immunoreactive neurons were distributed throughout all layers, especially in layers V and VI. Immunoreactivity was expressed by neurons belonging to different morphological classes: All CBPs were expressed in non-pyramidal neurons, but CB and CR were also found in pyramidal neurons.

**Discussion:**

The morphological characteristics of pyramidal and non-pyramidal neurons in the dolphin entorhinal cortex are similar to those described in the entorhinal cortex of other species, including primates and rodents. Interestingly, in primates, rodents, and dolphins, most of the CBP-containing neurons are found in the superficial layers, but the large CR-ir neurons are also abundant in the deep layers. Layers II and III of the entorhinal cortex contain neurons that give rise to the perforant pathway, which conveys most of the cortical information to the hippocampal formation. From the hippocampal formation, reciprocal projections are directed back to the deep layer of the entorhinal cortex, which distributes the information to the neocortex and subcortical area. Our data reveal that in the dolphin entorhinal cortex, the three major CBPs label morphologically heterogeneous groups of neurons that may be involved in the information flow between entorhinal input and output pathways.

## 1 Introduction

Dolphins have very large brains, making their Encephalization Quotient (EQ) comparable to that of many non-human primates (Jerison, 1973; Morgane et al., 1985; Marino, 2002; Marino et al., 2004, 2007). The increase in brain size is a result of selective pressures imposed by the aquatic environment on motor, sensory and eventually social capabilities (Marino, 2002; Marino et al., 2004, 2007). Although cetacean brain are very large, their entorhinal cortex, a constituent of the periarchicortex, is significantly reduced (Jacobs et al., 1971, 1979; Morgane and Jacobs, 1972; Morgane et al., 1980, 1986; Cozzi et al., 2017). The entorhinal cortex of terrestrial mammals comprises two main cytoarchitectonic subdivision in primates and rodents: the medial entorhinal cortex (MEA) and the lateral entorhinal cortex (LEA). These areas have a fourth layer, the *lamina dissecans*, which is essentially acellular and bears little homology with the layer IV found in the neocortex (Insausti et al., 1995, 1997; Krimer, 1997; Kerr et al., 2007; Insausti and Amaral, 2008; Witter, 2012; Cappaert Van Strien and Witter, 2015; Witter et al., 2017). The evidence from studies in terrestrial mammals, including non-human primates and rodents, shows a general pattern of connectivity and contribution of this cortical region to behavior that can be considered general for all mammals, including cetaceans. In terrestrial mammals the entorhinal cortex is the main entry point for the information processed by the hippocampal formation and provides the main conduit for processed information to be relayed back to the neocortex. In addition, the entorhinal cortex serves as the entry site for the projections directed towards the limbic complex, originating from the amygdala, the neocortex, and the olfactory bulb (Amaral et al., 1987; Carboni et al., 1990; Insausti, 1993; Insausti et al., 1997; Kerr et al., 2007; Insausti and Amaral, 2008, 2012; Witter, 2012; Cappaert Van Strien and Witter, 2015; Maass et al., 2015; Witter et al., 2017). The entorhinal cortex has been shown to be involved in high-level cognitive functions in terrestrial mammals, so understanding of its structural organization in cetaceans is particularly important given the extensive evidence for their cognitive abilities. The entorhinal cortex of the bottlenose dolphin occupies an area within the parahippocampal gyrus (Jacobs et al., 1979; Hof et al., 2005; Hof and Van Der Gucht, 2007). In particular, Jacobs et al. (1979) described a distinct six-layered entorhinal cortex with an extensive *lamina dissecans* and similar patterns to primates (figures 66 to 69), but with less extensive corticoperforant fibers bordering the archicortex [see also Breathnach and Goldby (1954)]. Direct experimental evidence of the connectivity of the dolphin entorhinal cortex is lacking, and thus, the functional significance of the cetacean entorhinal cortex can only be elucidated by comparison with other mammals. The organization of the entorhinal cortex can be studied using a variety of approaches. Most of the cytoarchitectural studies are performed using Nissl staining, which provides information about the organization and layering patterns of the cortex and on the basic morphology of the neurons. However, neurons with comparable morphology can be characterized by their variable neurochemical profile and thus by different functions. Therefore, it is important to combine morphological and neurochemical studies to obtain a more precise overview of the organization of the entorhinal cortex in a given species. Several neurochemical markers have been used to identify the neurochemical organization of the entorhinal cortex in rodents and primates, and among the most commonly used are the calcium-binding proteins (CaBPs) such as calretinin (CR), calbindin-D28k (CB), and parvalbumin (PV). These studies show that immunoreactivity for these three types of CABPs is observed in both excitatory (CR and CB) and inhibitory neurons (CR, CB, and PV) (Tuñón et al., 1992; Schmidt et al., 1993; Seress et al., 1994; Wouterlood et al., 1995, 2000; Fujimaru and Kosaka, 1996; Miettinen et al., 1996, 1997; Mikkonen et al., 1997; Berger et al., 1999; Suzuki and Porteros, 2002; Grateron et al., 2003; Kobro-Flatmoen and Witter, 2019). On the contrary, there is a lack of information on the distribution and morphology of CaBPs-immunoreactive (IR) neurons in the cetacean entorhinal cortex. In the present study, we investigated the cytoarchitecture of the entorhinal cortex of the bottlenose dolphin, perhaps the most studied cetacean species and a paradigm for dolphins and other small cetaceans. The distribution of CBP-immunoreactive neurons (CR-ir, CB-ir and PB-ir, respectively) helped us to map and define the organization of the area. The present data on the dolphin entorhinal cortex provide the basis for comparison with that of other mammals.

## 2 Materials and methods

### 2.1 Dolphin tissues

Dolphin brains (Table 1) were extracted during routine necropsy performed at the Department of Comparative Biomedicine and Food Science (BCA) of the University of Padova (Italy) on specimens. The brains were consequently fixed in phosphate buffered paraformaldehyde (4%), cut in coronal slices (about 1.5 cm × 2.5 cm) and stored in the *Mediterranean marine mammal tissue bank* (MMMTB, http://www.marinemammals.eu), located in BCA. The MMMTB is a CITES recognized (IT020) research center of the University of Padova, sponsored by and collaborating with the Italian Ministry of the Environment. MMMTB collects and stores samples from wild or captive marine mammals whose samples or whole carcasses are delivered to BCA for post-mortem diagnostics. Smaller blocks, containing the entorhinal cortex, were cut from the thick formalin-fixed tissue slices, washed in phosphate buffered saline (PBS) (pH 7.4), cryoprotected in 20% glycerol in 0.02 M potassium phosphate buffered saline (PBS) (pH 7.4) at +4° C for 48 h, frozen in dry ice, and stored at −70°C. Fifty-μm–thick frozen coronal sections (one-in-eight series) throughout the entire rostrocaudal extent of the entorhinal cortex were cut with a sliding microtome. The angle of coronal sectioning performed in this study was perpendicular to the surface of the entorhinal cortex. For immunohistochemical staining, the sections were stored in tissue-collecting solution (30% ethylene glycol, 25% glycerin in 0.05 M sodium phosphate buffer, pH 7.4) at −20°C. Another series of sections to be stained with thionin was stored in 10% formalin.

**Table 1:** Detail of the sampled bottlenose dolphins.

Specimen	ID	SEX	Origin	Length/Weight	Age
*T. truncates*	192	F	Stranded	240 cm/178.5 kg	Adult
	196	M	Stranded	300 cm/219 kg	Adult
	203	M	Stranded	284 cm/288 kg	Adult
	319	M	Stranded	310 cm	Adult

### 2.2 Thionin staining

To evaluate the boundaries and the layer-specific neurons of the entorhinal cortex, sections adjacent to immunoperoxidase sections were stained with thionin as follows. Sections were taken out of the 10% formaldehyde solution, mounted on gelatin-coated slides and dried overnight at 37°C. Sections were defatted 1 h in a mixture of chloroform/ethanol 100% (1.1), and then rehydrated through a graded series of ethanol, 2 × 2 min in 100% ethanol, 2 min in 96% ethanol, 2 min in 70% ethanol, 2 min in 50% ethanol, 2 min in dH2O, and stained 30 s in a 0.125% thionin (Fisher Scientific) solution, dehydrated and coverslipped with Entellan (Merck, Darmstaldt, Germany).

### 2.3 Immunoperoxidase

Three of the one-in-eight series of free-floating sections were collected from tissue-collecting solution and washed three times (10 min each) in 0.02 M phosphate buffer containing 0.9% sodium chloride (PBS; pH 7.4). To reduce the endogenous peroxidase activity, the sections were incubated in 3% hydrogen peroxide and 10% methanol in PBS for 30 min at room temperature. Nonspecific binding was blocked by incubating sections in a solution (0.5% Triton X-100 in PBS) containing 10% normal horse serum (NHS) for parvalbumin and calbindin immunohistochemistry or normal goat serum (NGS) for calretinin immunohistochemistry for 3 h at room temperature. The primary antibody incubations were done at 4°C for 2 to 3 days in a solution (0.5% Triton X-100 in PBS) containing either 1% NHS and monoclonal mouse anti-parvalbumin (dilution 1:3000, #235, Swant, Bellinzona, Switzerland) or monoclonal mouse anti–calbindin-D28k (dilution 1:3000, #McAB300, Swant), or 1% NGS and polyclonal rabbit anti-calretinin (dilution 1:3000, #7696, Swant, Bellinzona, Switzerland). After three washes (10 min each) in PBS containing either 2% NHS (parvalbumin and calbindin immunohistochemistry) or 2% NGS (calretinin immunohistochemistry), the sections were incubated in the secondary antibody solution (0.3% Triton X-100 in PBS) containing either biotinylated horse anti-mouse immunoglobulin G with 1% NHS (parvalbumin and calbindin; dilution 1:200, #BA-2,000, Vector, Burlingame, CA) or biotinylated goat anti-rabbit immunoglobulin G with 1% NGS (calretinin; dilution 1:200, #BA-1,000, Vector) for 2 h at room temperature. Sections were then washed twice as described above and incubated for 45 min at room temperature in avidin-biotin solution (BioStain SuperABC #11–001, Biomeda, Foster City, CA) in PBS. Thereafter, the sections were washed three times and reacted with 3,38-diaminobenzidine (0.05%) containing hydrogen peroxide (0.04%) in KPBS. After three washes, the sections were mounted onto gelatin-coated slides and dried overnight at 37°C.

### 2.4 Specificity of the antibodies

The amino acid sequence of the proteins investigated in this article of bottlenose dolphin (*Tursiops truncatus*) were compared with those of other mammals (and especially the rat). For this aim we used the Ensemble genomic database 1. The sequence of CB and CR is shared for over 93%, whereas correspondence for Gng2 and PV is over 70%. The specificity of the immuno-histochemical staining was tested in repeated trials as follows: substitution of either the primary antibody, the anti-rabbit or anti-mouse IgG, or the ABC complex by PBS or non-immune serum. Under these conditions the staining was abolished.

### 2.5 Analysis of sections

Sections stained using thionin and immunoperoxide were analyzed using an optical microscope (Axiophot, Zeiss, Germany). Brightfield images were recorded with a digital camera (AxioCam ERc5s^®^, Zeiss, Germany). The distribution of CR, CB, and PV-IR cell bodies in the LEA and MEA were plotted bilaterally in every fifth section throughout the enthorinal cortex with a computer-aided digitizing system (AccuStage 5.1, St. Shoreview, MN). Camera lucida drawings from the adjacent thionin-stained sections were used to define the laminar and regional boundaries of the areas of the entorhinal cortex. The outlines were superimposed on computer-generated plots using Corel Draw X3 (Corel Corporation, Ottawa, Ontario, Canada). AxioVision Rel.4.8 software (Zeiss) was utilized for morphometrical and morphological analysis of the thionin-stained and CR-, CB-, and PV-IR neurons in the LEA and MEA. In particular, for each animal, the perikaryal areas of thionin-stained and IR cell bodies of four non-consecutive sections of each entorhinal area were measured after manual tracing of the cell bodies outline. These morphometrical analyzes were done in each separate layer, with the exception of lamina dissecans, because of its low cellular density. Data were expressed as mean ± standard deviation (SD). Analysis of variance (ANOVA) was used to analyze whether there was any difference in the perikaryal main area of different IR cell types. The Tukey HSD *post hoc* test was used to make pair-wise comparisons between means. In the thionin-stained sections, cortical layers thicknesses were measured using the AxionVision Rel.4.8 software (Zeiss), using a tool measuring the length perpendicular to a line placed on the pial surface of the cortex. Measurements were made at least 5 times per sample, outside of sulcus bottom or top to avoid distortions. Contrast and brightness were adjusted to reflect the appearance of the labeling seen through the microscope using Adobe Photoshop CS3 Extended 10.0 software (Adobe Systems, San Jose, CA).

## 3 Results

### 3.1 Thionin staining: laminar and regional organization of the entorhinal cortex

#### 3.1.1 Laminar organization

**Figure 1:**
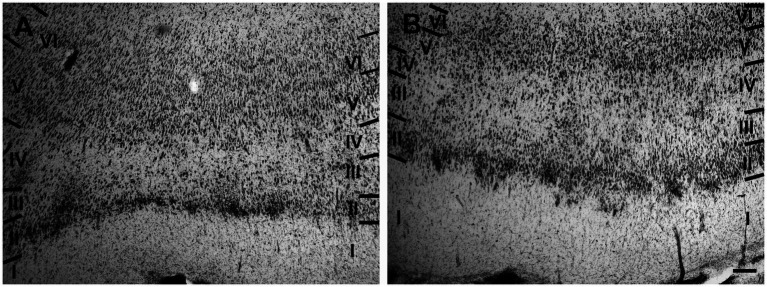
Brightfield photomicrographs of thionin-stained coronal sections from lateral entorhinal cortex (LEA) **(A)** and medial entorhinal cortex (MEA) **(B)**. Scale bar = 200 μm in B [applied to **(A)** and **(B)**].

The entorhinal cortex was located ventrocaudally to the amygdaloid complex and the hippocampal formation and has been divided into two main areas: lateral entorhinal area (LEA) and medial entorhinal area (MEA). Six layers were identified in the entorhinal cortex (LEA and MEA): molecular layer (layer I), stellate cell layer (layer II), superficial pyramidal cell layer (Layer III), *lamina dissecans* (layer IV), deep pyramidal cell layer (layer V), and polymorph cell layer (layer IV; Figure 1).

**Figure 2:**
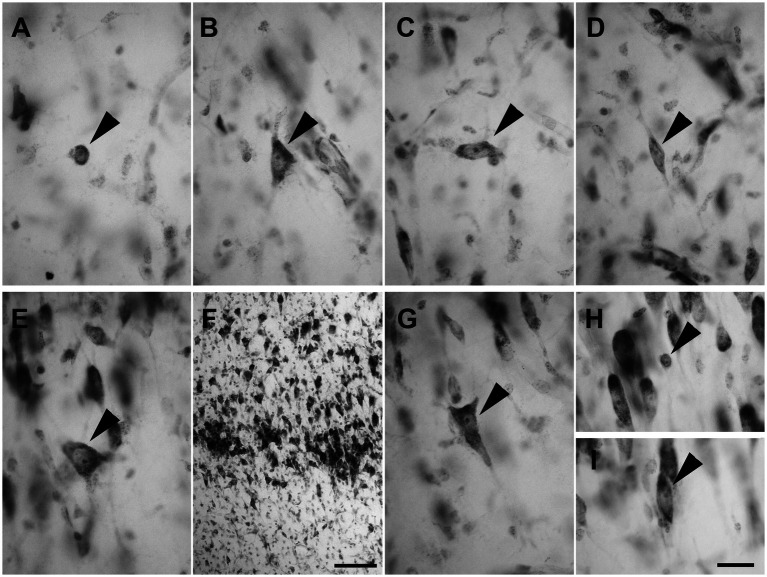
Brightfield photomicrographs of thionin-stained coronal sections from layers I **(A–D)** and II **(E–H)** of the lateral entorhinal cortex (LEA) and medial entorhinal cortex (MEA). Layer I contains spheroidal [arrowhead in **(A)**, LEA], polygonal [arrowhead in **(B)**, MEA], and fusiform [arrowhead in **(C)**, LEA; **(D)** MEA] nonpyramidal neurons. Fusiform neurons are oriented horizontally [arrowhead in **(C)**] or vertically [arrowhead in **(D)**] with respect to the cortical surface. Layer II shows darkly stained neurons with a polygonal cell body [arrowhead in **(E)**, LEA], usually aggregated into “islands” [arrowhead in **(F)**, LEA]. Layer II shows pyramidal neurons with the apical dendrite directed toward to the cortical surface [arrowhead in **(G)**, MEA], spheroidal neurons [arrowhead in panel **(H)**, LEA], and medium-sized fusiform cells [arrowhead in **(I)**, MEA]. Scale bar = 100 μm in **(F)**; 20 μm in **(H)** [applied to **(A–E)** and **(G–I)**].

Layer I was populated by a small number of sparse spheroidal (Figure 2A; *n* = 67 in LEA; *n* = 61 in MEA), polygonal (Figure 2B; *n* = 69 in LEA; *n* = 64 in MEA), and fusiform (Figures 2C,D; *n* = 78 in LEA; *n* = 75 in MEA) neurons of small size. Fusiform neurons were oriented horizontally (Figure 2C) or vertically (Figure 2D) with respect to the cortical surface.

Layer II contained darkly stained neurons with a polygonal soma (Figure 2E; *n* = 170 in LEA; *n* = 178 in MEA). Polygonal neurons were usually aggregated into “islands” (Figure 2F). Pyramidal neurons with the apical dendrite directed to the cortical surface could be observed (Figure 2G; *n* = 1,228 in LEA; *n* = 1,136 in MEA). Layer II also showed small spheroidal neurons (Figure 2H; *n* = 361 in LEA; *n* = 354 in MEA) and medium-sized fusiform cells (Figure 2I; *n* = 114 in LEA; *n* = 111 in MEA).

**Figure 3:**
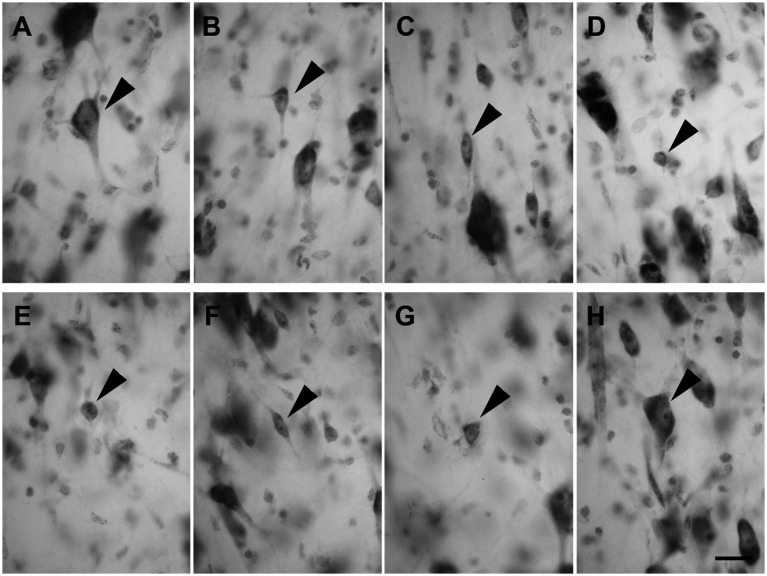
Brightfield photomicrographs of thionin-stained coronal sections of layers III **(A–D)** and IV **(E–H)** of the lateral entorhinal cortex (LEA) and medial entorhinal cortex (MEA). Layer III is composed of pyramidal neurons [arrowhead in **(A)**, LEA] and non-pyramidal neurons with a polygonal [arrowhead in **(B)**, MEA], fusiform [arrowhead in **(C)**, LEA], or spheroidal somata [arrowhead in **(D)**, MEA]. Layer IV (*lamina dissecans*) contains spheroidal [arrowhead in **(E)**, LEA], fusiform [arrowhead in **(F)**, LEA], polygonal [arrowhead in **(G)**, LEA], and pyramidal neurons [arrowhead in **(H)**, LEA]. Scale bar = 20 μm in **(H)** [applied to **(A–H)**].

Layer III was composed of a wide variety of neurons with a pyramidal (Figure 3A; *n* = 2,337 in LEA; *n* = 2,298 in MEA), polygonal (Figure 3B; *n* = 391 in LEA; *n* = 389 in MEA), fusiform (Figure 3C; *n* = 408 in LEA; *n* = 391 in MEA), or spheroidal cell bodies (Figure 3D; *n* = 205 in LEA; *n* = 197 in MEA). Pyramidal neurons were the most numerous and appeared densely packed in the inner part of the layer.

Layer IV (*lamina dissecans*) contained rare spheroidal (Figure 3E), fusiform (Figure 3F), and polygonal (Figure 3G) neurons with a small soma. However, darkly stained pyramidal neurons were observed (Figure 3H).

**Figure 4:**
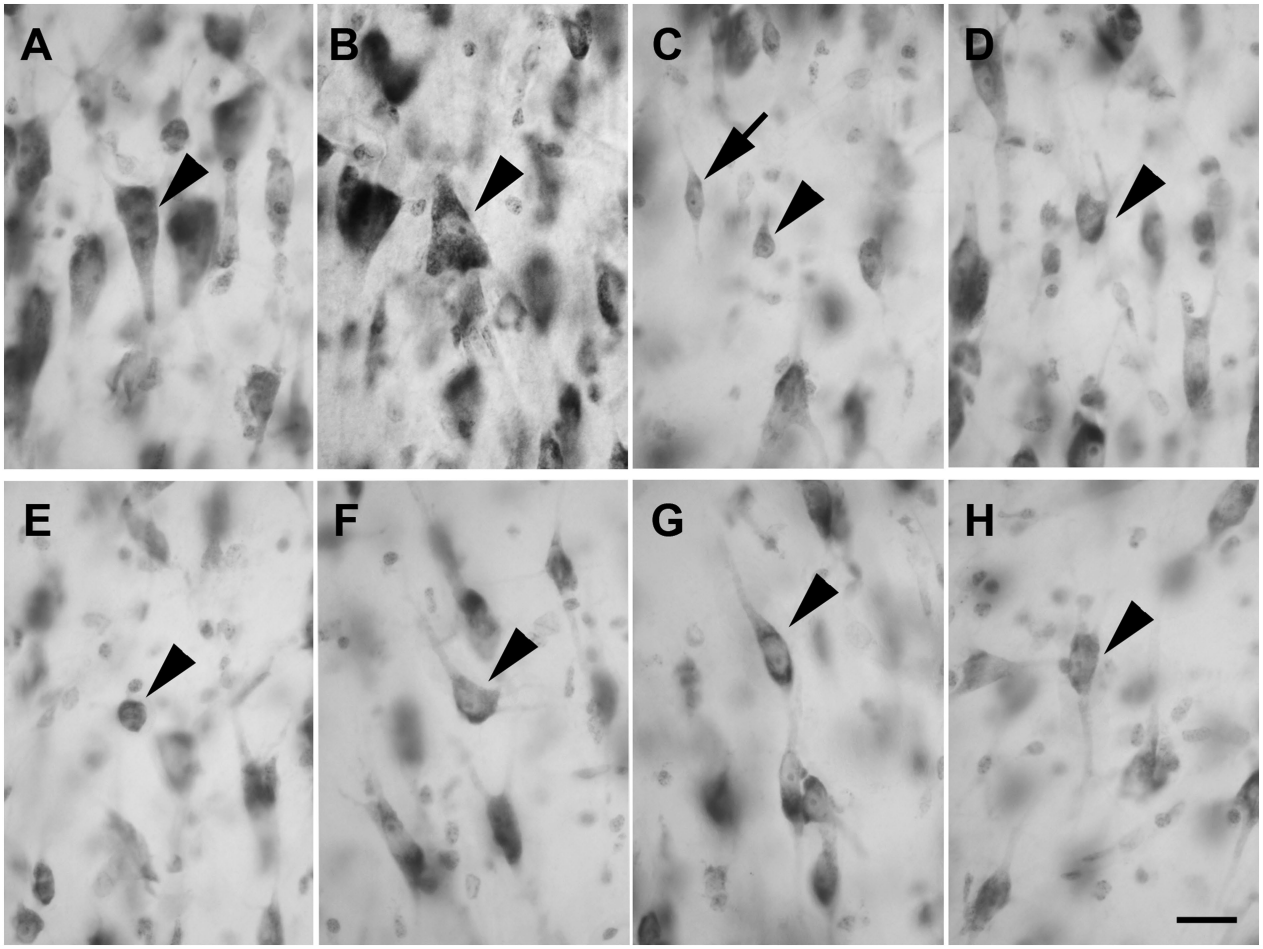
Brightfield photomicrographs of thionin-stained coronal sections from layers V **(A–D)** and VI **(E–H)** of the lateral entorhinal cortex (LEA) and medial entorhinal cortex (MEA). Layer V shows large pyramidal neurons [arrowhead in **(A)**, LEA]; interestingly, many inverted pyramidal cells are also present [arrowhead in **(B)**, MEA]. Layer V contains non-pyramidal neurons with a spheroidal [arrowhead in **(C)**, LEA], fusiform [arrow in **(C)**, LEA], and polygonal [arrowhead in **(D)**, MEA] cell bodies. Layer VI contains neurons with a spheroidal [arrowhead in **(E)**, LEA], polygonal [arrowhead in **(F)**, MEA], fusiform [arrowhead in **(G)**, LEA], and pyramidal [arrowhead in **(H)**, MEA] morphology. Scale bar = 20 μm in H [applied to **(A–H)**].

Layer V showed large and darkly stained pyramidal neurons (Figure 4A; *n* = 3,528 in LEA; *n* = 3,478 in MEA); interestingly, many inverted pyramidal cells were also observed (Figure 4B; *n* = 534 in LEA; *n* = 528 in MEA). Lightly stained neurons with a spheroidal (Figure 4C; *n* = 237 in LEA; *n* = 240 in MEA), fusiform (Figure 4C; *n* = 461 in LEA; *n* = 432 in MEA) or polygonal (Figure 4D; *n* = 418 in LEA; *n* = 402 in MEA) morphology could be observed among the pyramidal cells.

Layer VI was composed of a variety of morphological cell types with different sizes and spheroidal (Figure 4E; *n* = 125 in LEA; *n* = 124 in MEA), polygonal (Figure 4F; *n* = 361 in LEA; *n* = 368 in MEA), fusiform (Figure 4G; *n* = 412 in LEA; *n* = 406 in MEA), and pyramidal (Figure 4H; *n* = 1,469 in LEA; *n* = 1,452 in MEA) morphology.

#### 3.1.2 Regional organization

##### 3.1.2.1 Lateral entorhinal area (LEA)

Layer I was thick. Layer II was narrower, the neurons stained darker than in MEA, and many neurons were densely packed and formed cell islands. Between layers II and III there was a clear zone of sparse cells. Neurons in layer III formed a continuous band. Layer IV (*lamina dissecans*) was clearly visible. Neurons of layer V were dispersed, whereas neurons of layer VI were more densely packed than in layer V (Figure 1A).

##### 3.1.2.2 Medial entorhinal area (MEA)

Layer I was very thick. Layer II neurons formed a discontinuous band and were larger and stain darken than neurons of layer III. Layer III was much wider than layer II and contained neurons with a small somata. Layer IV (*lamina dissecans*) was not very clearly visible. Layer V contained large neurons, whereas layer VI exhibited smaller and more densely packed neurons than layer V. Layers V and VI were thinner than in LEA (Figure 1B).

**Figure 5:**
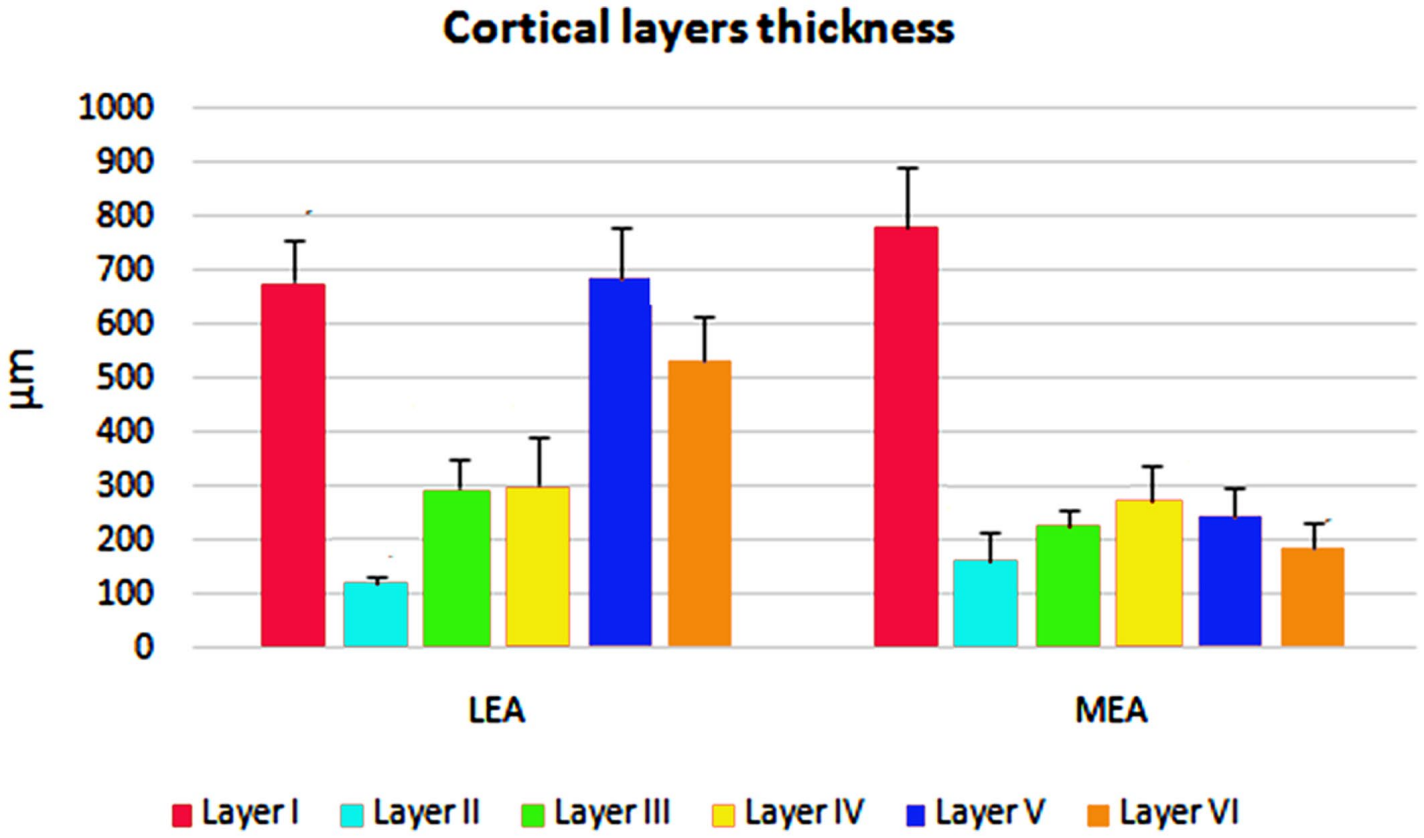
Cortical layers thicknesses ± standard deviation (SD) in lateral entorhinal area (LEA) and medial entorhinal area (MEA) of bottlenose dolphin.

**Figure 6:**
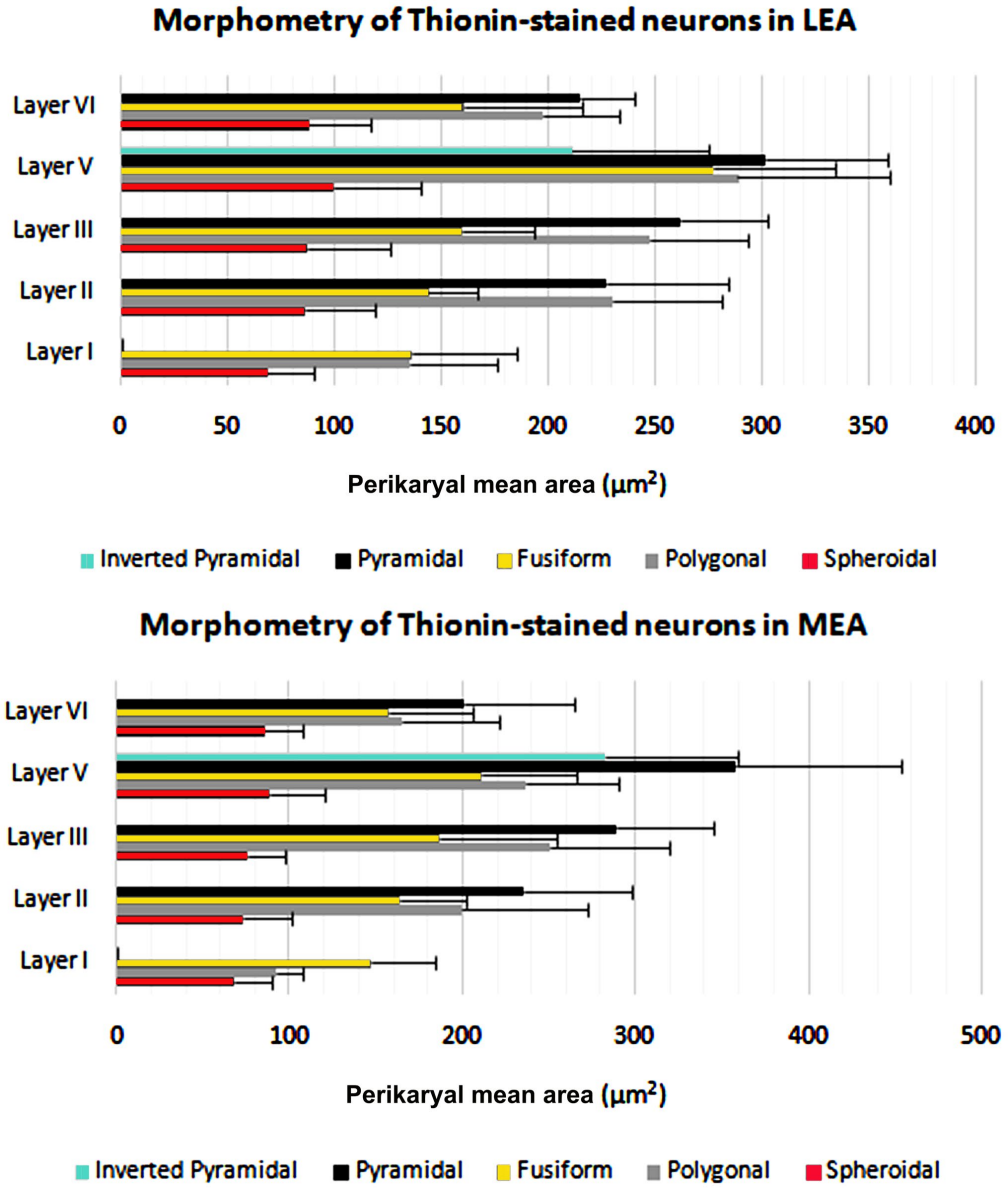
Perikaryal mean area ± standard deviation (SD) of thionin-stained neurons in lateral entorhinal area (LEA) and medial entorhinal area (MEA) of bottlenose dolphin.

The cortical layer thickness and the morphometric properties of neurons in LEA and MEA are shown in Figures 5, 6.

### 3.2 Calcium-binding proteins in the entorhinal cortex

**Figure 7:**
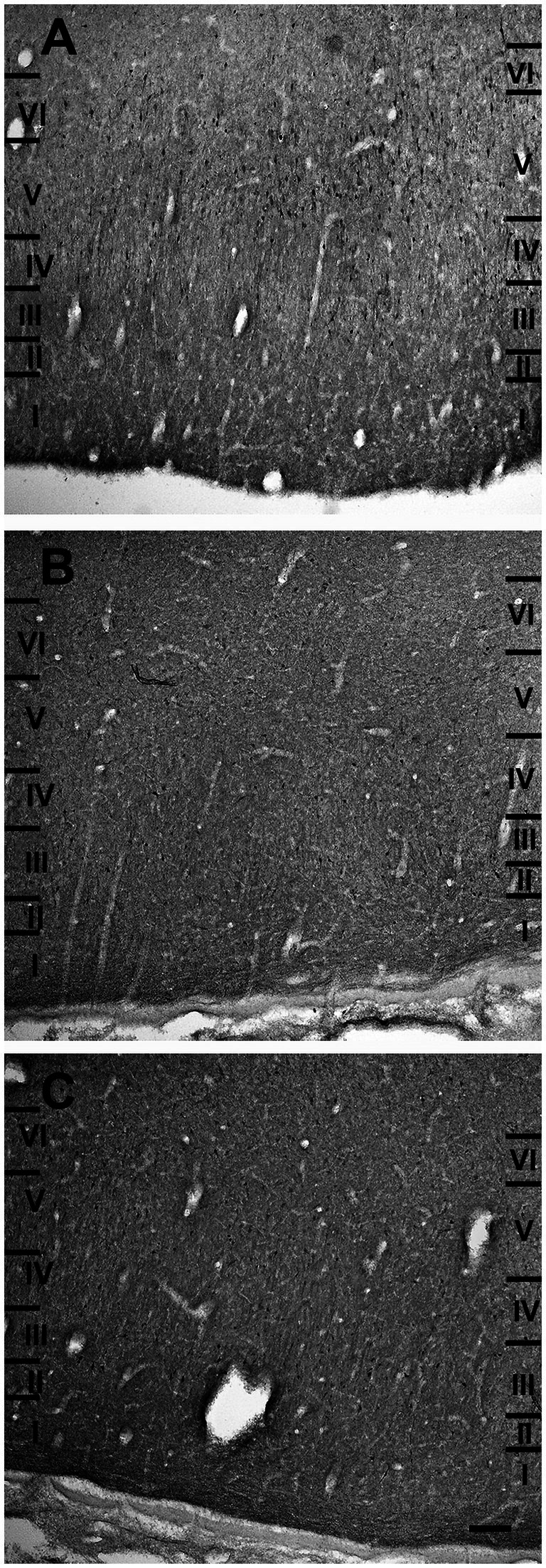
Brightfield photomicrographs of immunohistochemically stained sections demonstrating the distribution of calretinin **(A)**, calbindin-D28k **(B)**, and parvalbumin **(C)** immunoreactivity in the lateral entorhinal cortex. Scale bar = 200 μm in **(C)** [applied to **(A–C)**].

**Figure 8:**
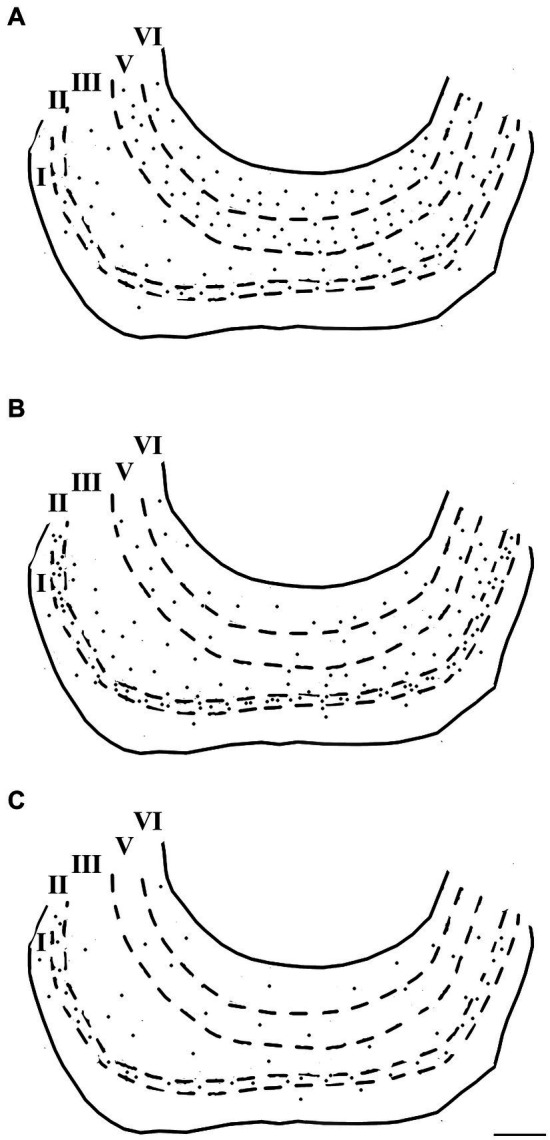
Computer-generated plots demonstrating the distribution of neurons immunoreactive for calretinin **(A)**, calbindin-D28k **(B)**, and parvalbumin **(C)** in the lateral entorhinal cortex. Each dot represents one immunopositive soma. Dashed lines delineate the different layers which are labeled with Roman numerals. Neurons in the putative layer IV were too sparse to be effectively represented here. Scale bar = 500 μm in **(C)** [applied to **(A–C)**].

Immunoreactivity for the three major calcium-binding proteins (CR, CB, and PV) showed a prominent laminar distribution in the dolphin entorhinal cortex (Figures 7, 8). Neurons immunostained for CR and, to a lesser extent, CB were prevalent, whereas PV was present in few neurons. The highest concentrations of PV-IR and CB-IR neurons were found in layers II and III, whereas a large number of neurons immunopositive for CR were found in the deep layers. In addition, most of the large CR-IR pyramidal cells were found in the deep layers, whereas most of the PV-IR non-pyramidal neurons were found in the superficial layers. The distribution of the immunoreactivities did not differ between LEA and MEA.

Neurons containing calcium-binding proteins were morphologically heterogeneous and could be divided into two main categories: pyramidal and non-pyramidal neurons. Non-pyramidal neurons could also subdivided in spheroidal, polygonal and fusiform cells.

#### 3.2.1 Pyramidal neurons

**Figure 9:**
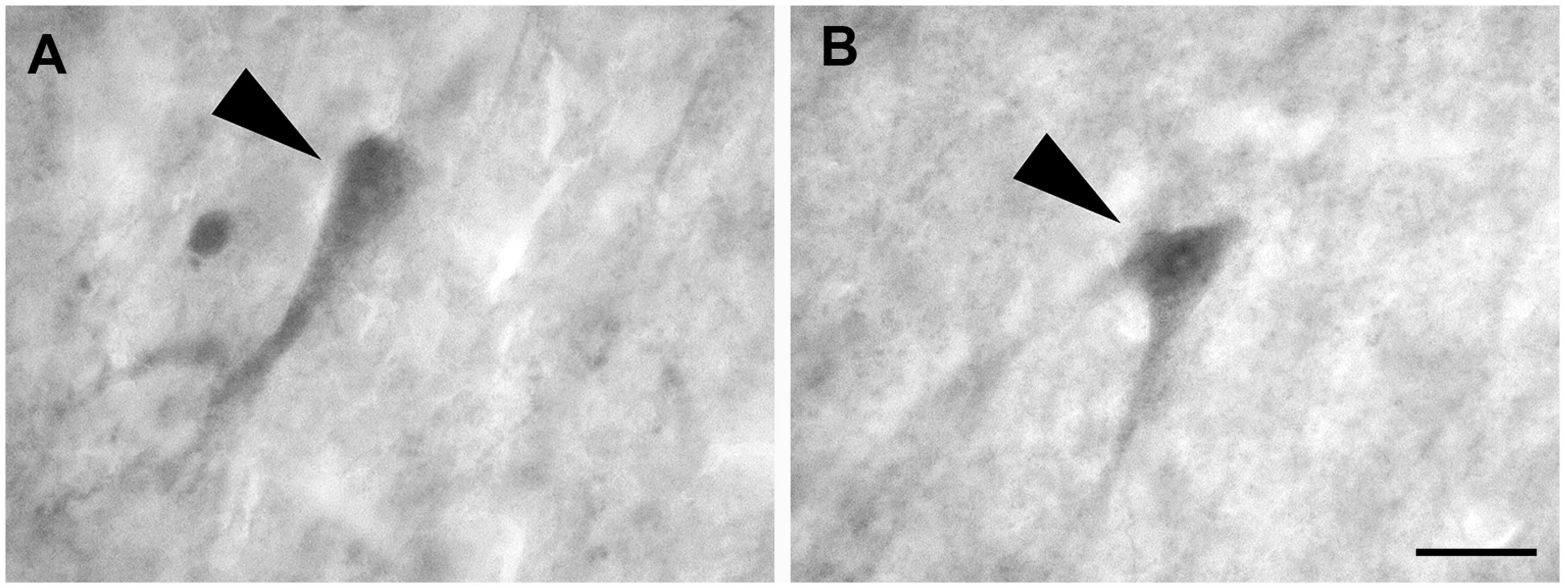
Brightfield photomicrographs demonstrating pyramidal neurons (arrowheads) immunoreactive for calretinin (layer V) **(A)** and calbindin-D28k (layer V) **(B)**. Scale bar = 20 μm in **(B)** [applied to **(A,B)**].

These cells, observed only in CB and CR preparations, had a pyramidal cell body from which the dendrites extended for only a short distance. The largest pyramidal neurons were observed in layers V and VI (Figures 9A,B).

**Figure 10:**
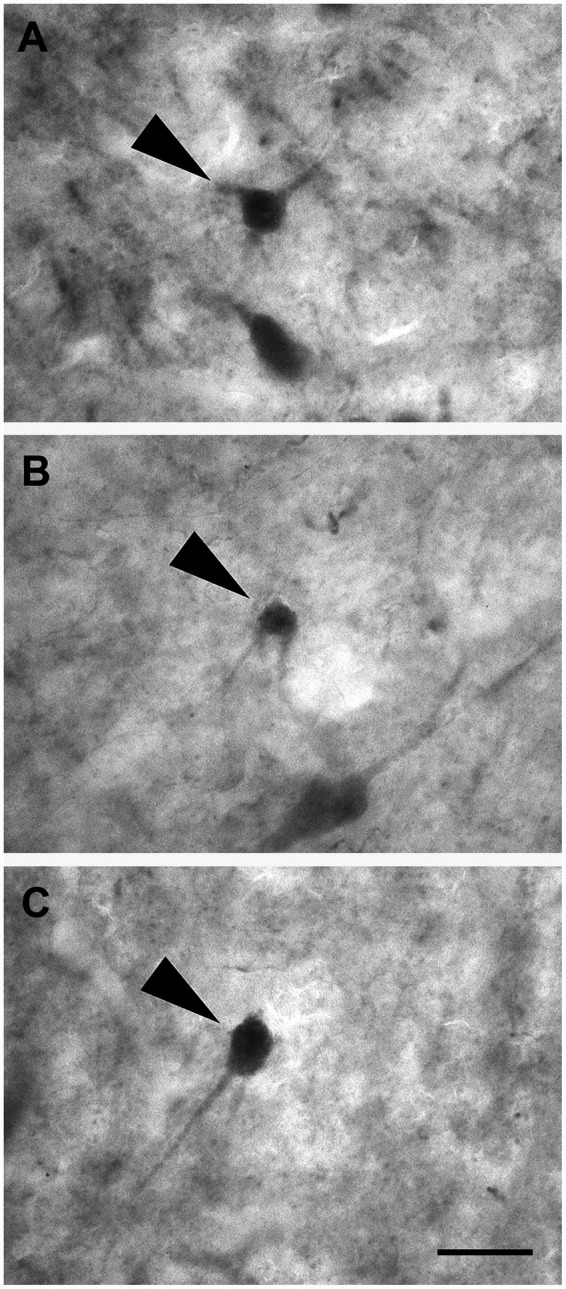
Brightfield photomicrographs demonstrating non-pyramidal spheroidal neurons (arrowheads) immunoreactive for calretinin (layer V) **(A)**, calbindin-D28k (layer III) **(B)**, and parvalbumin (layer II) **(C)**. Scale bar =20 μm in **(C)** [applied to **(A–C)**].

Non-pyramidal spheroidal neurons: These neurons had a small spheroidal cell body that gave rise to a few thin dendrites (Figures 10A–C).

#### 3.2.2 Non-pyramidal polygonal neurons

**Figure 11:**
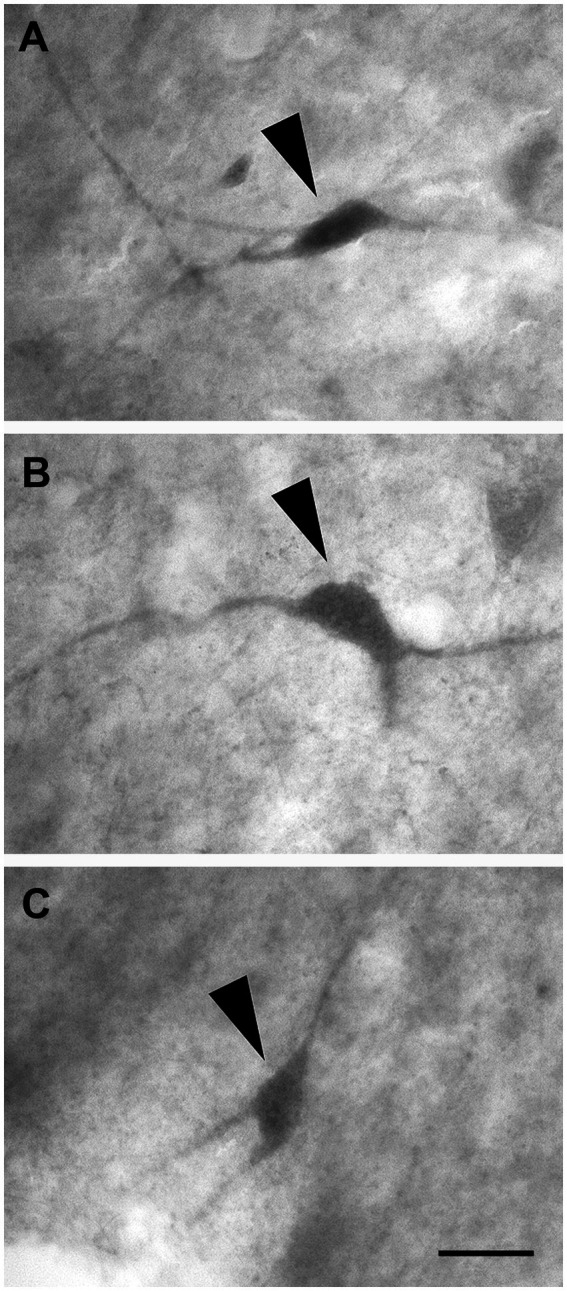
Brightfield photomicrographs demonstrating non-pyramidal polygonal neurons (arrowheads) immunoreactive for calretinin (layer V) **(A)**, calbindin-D28k (layer III) **(B)**, and parvalbumin (layer III) **(C)**. Scale bar = 20 μm in **(C)** [applied to **(A–C)**].

These cells had a polygonal soma of variable size and giving rise to several dendrites of varying thickness (Figures 11A–C).

#### 3.2.3 Non-pyramidal fusiform neurons

**Figure 12:**
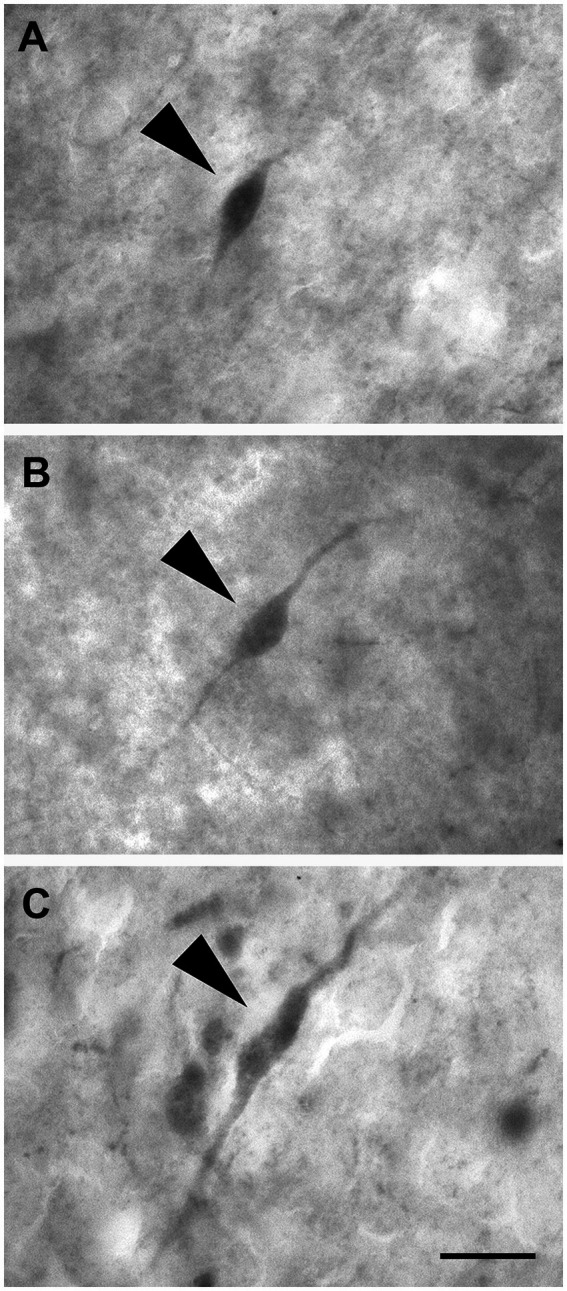
Brightfield photomicrographs demonstrating nonpyramidal fusiform neurons (arrowheads) immunoreactive for calretinin (layer III) **(A)**, calbindin-D28k (layer II) **(B)**, and parvalbumin (layer II) **(C)**. Scale bar = 20 μm in **(C)** [applied to **(A–C)**].

These neurons had two dendrites emerging from opposite poles of fusiform cell bodies. The dendrites often branched near the somata (Figures 12A–C).

**Figure 13:**
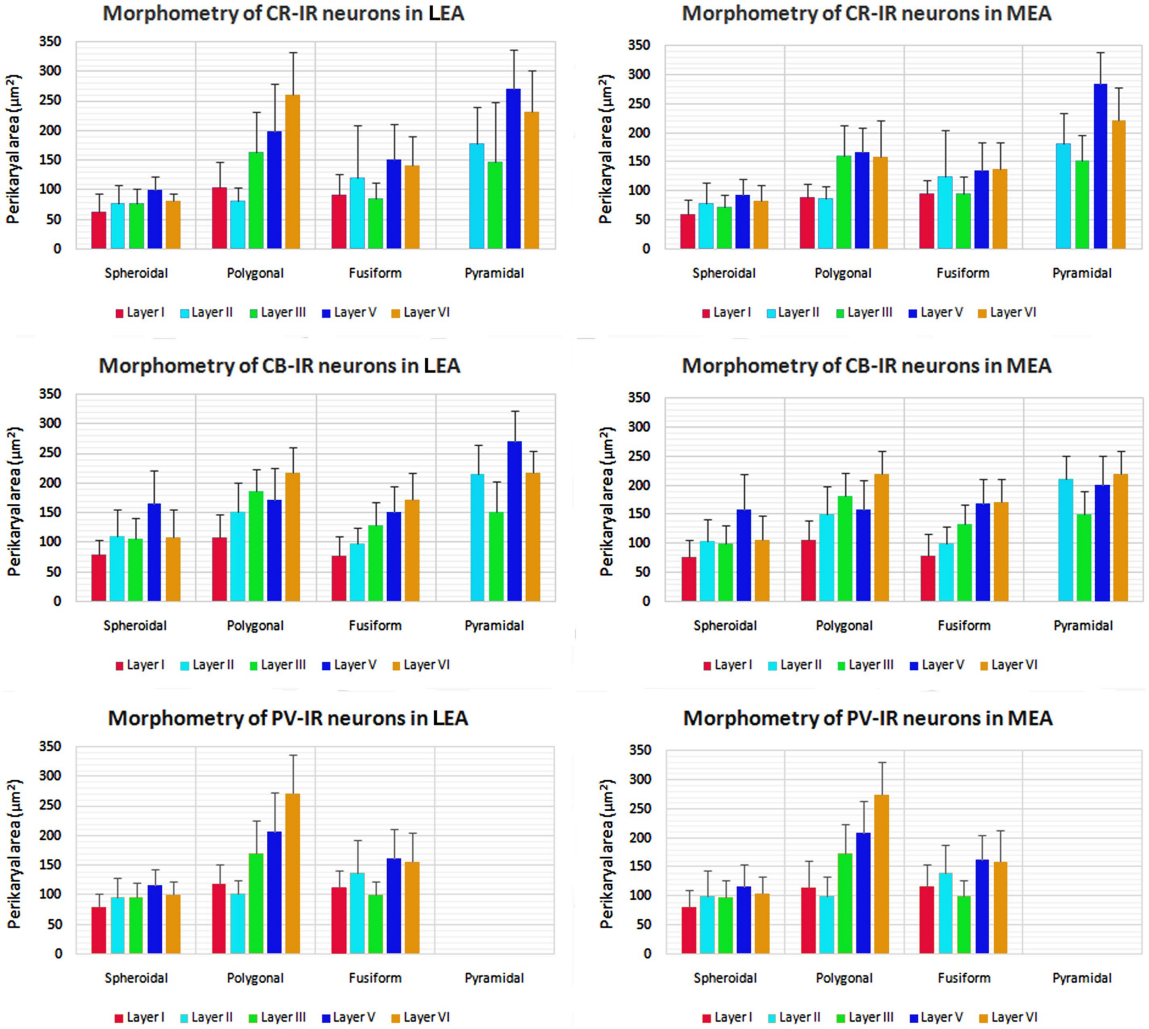
Perikaryal mean area ± standard deviation (SD) of calretinin- (CR), calbindin-D28k (CB), and parvalbumin- (PV) immunoreactive neurons in lateral entorhinal area (LEA) and medial entorhinal area (MEA) of bottlenose dolphin. For the explanations see the text.

The morphometric characteristics of calcium-binding proteins-IR neurons in LEA and MEA are reported in Figure 13. The morphometric features of IR neurons were not statistically different when comparing LEA with MEA. With the exception of CB-IR neurons located in layer V, the mean perikaryal area of the CBPs-IR polygonal neurons was significantly larger than that of spheroidal neurons (*p* < 0.001; Figure 13). Also, the mean perikaryal area of pyramidal neurons immunoreactive for the CR and CB was larger than that of other cell types. In layer II of LEA and MEA, pyramidal neurons IR for the CR are statistically smaller than those IR for CB (*p* < 0.001; Figure 13), whereas the opposite was observed in layers V and VI (*p* < 0.001; Figure 13). Throughout the entorhinal cortex, the perikaryal size of the CR-IR nonpyramidal neurons was statistically smaller than that of nonpyramidal neurons immunoreactive for CB and PV (*p* < 0.001; Figure 13).

### 3.3 Laminar distribution of calcium-binding proteins immunoreactive neurons in the entorhinal cortex

#### 3.3.1 Layer I

##### 3.3.1.1 CR

Layer I contained some small or medium-sized CR-IR spheroidal (*n* = 15 in LEA; *n* = 13 in MEA), polygonal (*n* = 16 in LEA; *n* = 14 in MEA), and fusiform neurons (*n* = 19 in LEA; *n* = 20 in MEA). Fusiform cells appeared bipolar with horizontally oriented thin dendrites.

##### 3.3.1.2 CB

Scattered spheroidal (*n* = 11 in LEA; *n* = 13 in MEA), polygonal (*n* = 9 in LEA; *n* = 12 in MEA) and fusiform (*n* = 9 in LEA; *n* = 10 in MEA) small or medium-sized CB-IR neurons were observed in layer I, their dendrites appeared mostly confined within the layer.

##### 3.3.1.3 PV

Occasionally PV-IR neurons with spheroidal (*n* = 5 in LEA; *n* = 4 in MEA), polygonal (*n* = 6 in LEA; *n* = 4 in MEA), and fusiform (*n* = 5 in LEA; *n* = 5 in MEA) somata were occasionally observed in layer I.

#### 3.3.2 Layer II

##### 3.3.2.1 CR

Small or medium sized CR-IR nonpyramidal neurons with spheroidal (*n* = 112 in LEA; *n* = 117 in MEA), polygonal (*n* = 51 in LEA; *n* = 47 in MEA) and fusiform (*n* = 33 in LEA; *n* = 39 in MEA) somata were located in layer II. Large CR-IR pyramidal cells were also observed (*n* = 68 in LEA; *n* = 65 in MEA).

##### 3.3.2.2 CB

Neurons immunoreactive for the CB were with a pyramidal (*n* = 51 in LEA; *n* = 57 in MEA) spheroidal (*n* = 83 in LEA; *n* = 87 in MEA), polygonal (*n* = 74 in LEA; *n* = 81 in MEA), and fusiform (*n* = 32 in LEA; *n* = 41 in MEA) somata. Pyramidal neurons were clustered and had an evident apical dendrite directed into layer I.

##### 3.3.2.3 PV

PV-IR neurons were non-pyramidal cells with a spheroidal (*n* = 41 in LEA; *n* = 47 in MEA), polygonal (*n* = 35 in LEA; *n* = 37 in MEA), and fusiform (*n* = 33 in LEA; *n* = 31 in MEA) morphology. Fusiform neurons showed an evident somata.

#### 3.3.3 Layer III

##### 3.3.3.1 CR

Layer III contained non-pyramidal cells with spheroidal (*n* = 62 in LEA; *n* = 70 in MEA), polygonal (*n* = 121 in LEA; *n* = 119 in MEA), and fusiform (*n* = 130 in LEA; *n* = 142 in MEA) somata of various sizes and medium-sized pyramidal cells (*n* = 107 in LEA; *n* = 106 in MEA).

##### 3.3.3.2 CB

CB-IR neurons located in layer III were morphologically similar to those observed in CR preparations and showed a spheroidal (*n* = 121 in LEA; *n* = 131 in MEA), polygonal (*n* = 54 in LEA; *n* = 58 in MEA), fusiform (*n* = 71 in LEA; *n* = 76 in MEA), and pyramidal (*n* = 44 in LEA; *n* = 50 in MEA) cell bodies.

##### 3.3.3.3 PV

PV-IR neurons in layer III had a polygonal (*n* = 52 in LEA; *n* = 56 in MEA) and, to a lesser extent, spheroidal (*n* = 38 in LEA; *n* = 39 in MEA) and fusiform (*n* = 40 in LEA; *n* = 41 in MEA) morphologies.

#### 3.3.4 Layer IV

Neurons immunoreactive for the three calcium-binding proteins with spheroidal, polygonal, and fusiform cell bodies were seldom observed in layer IV.

#### 3.3.5 Layer V

##### 3.3.5.1 CR

Layer V contained medium-sized non-pyramidal neurons with spheroidal (*n* = 74 in LEA; *n* = 63 in MEA), polygonal (*n* = 131 in LEA; n = 141 in MEA) or fusiform (*n* = 150 in LEA; *n* = 152 in MEA) cell bodies and many pyramidal neurons (*n* = 167 in LEA; *n* = 157 in MEA) with a large somata.

##### 3.3.5.2 CB

Few CB-IR neurons were observed in layer V as compared with layers II and III. These cells could be large pyramidal (*n* = 13 in LEA; *n* = 17 in MEA) and non-pyramidal neurons with spheroidal (*n* = 51 in LEA; *n* = 47 in MEA), polygonal (*n* = 63 in LEA; *n* = 68 in MEA) and fusiform (*n* = 37 in LEA; *n* = 35 in MEA) cells bodies. Pyramidal cells are usually smaller than those observed in the CR preparation.

##### 3.3.5.3 PV

The few PV-IR neurons observed in layer V were only medium-sized nonpyramidal cells with a spheroidal (*n* = 27 in LEA; *n* = 25 in MEA), polygonal (*n* = 33 in LEA; *n* = 34 in MEA), and fusiform (*n* = 35 in LEA; *n* = 32 in MEA) cell bodies.

#### 3.3.6 Layer VI

##### 3.3.6.1 CR

Layer VI contained many large CR-IR pyramidal neurons (*n* = 62 in LEA; *n* = 70 in MEA) with an evident apical dendrite. In addition, layer VI contained medium-sized spheroidal (*n* = 62 in LEA; *n* = 70 in MEA), polygonal (*n* = 62 in LEA; *n* = 70 in MEA), and fusiform (*n* = 62 in LEA; *n* = 70 in MEA) non-pyramidal cells.

##### 3.3.6.2 CB

Layer VI showed the same morphologic types of CB-IR neurons as reported in CR preparation: spheroidal (*n* = 40 in LEA; *n* = 39 in MEA), polygonal (*n* = 63 in LEA; *n* = 73 in MEA), fusiform (*n* = 11 in LEA; *n* = 13 in MEA) and pyramidal (*n* = 14 in LEA; *n* = 17 in MEA) neurons. However, the pyramidal cells immunoreactive for the CB are smaller than those positive for CR.

##### 3.3.6.3 PV

PV-IR non-pyramidal neurons had a medium sized somata with a spheroidal (*n* = 23 in LEA; *n* = 23 in MEA) and fusiform (*n* = 33 in LEA; *n* = 34 in MEA) morphology. Interestingly, large polygonal non-pyramidal cells (*n* = 31 in LEA; *n* = 33 in MEA) were also observed.

## 4 Discussion

In recent years, research on the neuroanatomical features of the bottlenose dolphin has increased (Bombardi et al., 2010, 2011, 2013, 2021; Cozzi et al., 2014; Parolisi et al., 2015; Rambaldi et al., 2017; Sacchini et al., 2018, 2022; Graïc et al., 2021, 2022; Gerussi et al., 2023). In particular, the precise topographical and functional identification of the dolphin neocortical areas, and their comparison with those of terrestrial mammals, is challenging. Previous studies have mapped the principal motor and sensory areas in common and bottlenose dolphins (Lende and Akdikmen, 1968; Morgane et al., 1980; Ridgway, 1990; Cozzi et al., 2017). A recent study identified the dolphin equivalent of the human prefrontal cortex in the bottlenose dolphin (Gerussi et al., 2023). Overall, however, considered in as a whole, several features of the dolphin brain remain poorly documented compared to other mammals. However, these studies were primarily cytoarchitectural determinations and did not report immunocytochemical characteristics of the neurons in the entorhinal and limbic regions. The entorhinal area is strongly connected with the hippocampal formation in terrestrial mammals, including Artiodactyls (Amaral et al., 1987; Carboni et al., 1990; Insausti, 1993; Insausti et al., 1997; Kerr et al., 2007; Insausti and Amaral, 2008, 2012; Witter, 2012; Cappaert Van Strien and Witter, 2015; Maass et al., 2015; Witter et al., 2017). The hippocampal formation of dolphins and whales is very small (Morgane et al., 1982; Oelschlager and Buhl, 1985; Oelschläger and Buhl, 1985; Morgane and Jacobs, 1986), leading many authors to propose that the organization of the central part of the limbic system differs significantly from that of terrestrial mammals. Since dolphins lack olfaction [for reference see Cozzi et al. (2017)], the absence of an olfactory bulb raises interest in the entorhinal cortex. In addition, the study of the entorhinal cortex in cetaceans is particularly interesting given the presence of a small hippocampal formation in these animals. In the present study, we report that the dolphin entorhinal cortex, as in terrestrial mammals, is composed of six layers, of which layer IV (*lamina dissecans*) contains rare and irregularly distributed neurons [for details on this entorhinal layer, reference and review see Insausti et al. (2017)]. The classical mammalian entorhinal cortex consists of two divisions: LEA and MEA. This bipartition has been widely used because of LEA and MEA can be easily distinguished by their respective distinct cytoarchitecture. Although several subsequent studies have shown that the entorhinal cortex of primates and rodents can be further partitioned (Insausti et al., 1997; Insausti and Amaral, 2008; Witter, 2012; Cappaert Van Strien and Witter, 2015; Witter et al., 2017; Piguet et al., 2018), here we utilized the traditional subdivision considering that the bottlenose dolphins is a relatively new species to describe. Specifically, our cytoarchitectural analysis shows that the two recognized subdivisions, LEA and MEA. can be easily identified in the bottlenose dolphin, as in terrestrial mammals (Kobro-Flatmoen and Witter, 2019). Layer II is more clearly demarcated in LEA than in MEA; in addition, the boundary between layers II and III is very sharp in LEA. The cell-sparse zone between layers II and III was named *external lamina dissencans* by Jacobs et al. (1979). The neurons in layer II of LEA are clustered in islands and smaller than in the MEA. The layer IV (*lamina dissecans*) of the the MEA is less distinct than in the LEA, whereas layers V (thicker in the LEA than in the MEA) and VI (thicker in the LEA than in the MEA) were slightly better differentiated from each other in the MEA than in the LEA. Overall, the general appearance of the dolphin entorhinal cortex is similar to that observed in terrestrial mammals; however, layer is characteristically thicker VI in the dolphin LEA than in other mammals (Morgane and Glezer, 1990). We also emphasize the importance of layer II in the thalamo-related circuitry of Artiodactyls (Peruffo et al., 2019), its role as a potential reservoir of immature neurons, and its progressive increase in neuronal density in large-brained species such as the bottlenose dolphin (La Rosa et al., 2020).

We also examined the distribution of CBPs in the entorhinal cortex of the bottlenose dolphin. CBPs, such as CR, CB, and PV have been observed in the entorhinal cortex of different species and found to be localized in morphologically distinct populations of neurons (Tuñón et al., 1992; Schmidt et al., 1993; Seress et al., 1994; Wouterlood et al., 1995, 2000; Fujimaru and Kosaka, 1996; Miettinen et al., 1996, 1997; Mikkonen et al., 1997; Berger et al., 1999; Suzuki and Porteros, 2002; Grateron et al., 2003; Kobro-Flatmoen and Witter, 2019). These proteins are also colocalize with γ-aminobutyric acid (GABA) and can be used as proxy markers of local circuit interneurons (Kobro-Flatmoen and Witter, 2019). CBPs have been studied in the dolphin brain (Glezer et al., 1992, 1993, 1995, 1998; Hof et al., 2000; Cozzi et al., 2014; Graïc et al., 2022), but information on their presence in the entorhinal cortex is limited. In our experimental series, CR-ir and CB-ir neurons were always easily identified. The majority of the CB-ir cells were confined to the superficial layers, whereas the CR-ir neurons were distributed throughout all the cortical columns of the entorhinal cortex. CR-ir and CB-ir neurons were far more present, and consequently distinct, than PV-ir neurons, which were localized mainly in superficial layers II and III. The data that we report here confirm numerous previous experiments using CBPs in different areas of cetacean brain (Hof et al., 1992, 2000; Glezer et al., 1993, 1995; Hof and Sherwood, 2005), as well as evidence showing that PV immunostaining was scarce or utterly absent in the cetacean cortex (Glezer et al., 1998; Cozzi et al., 2014; Graïc et al., 2021).

Immunoreactivity for the CR and the CB neurons was observed in both pyramidal and non-pyramidal neurons, but the PV was only expressed only in non-pyramidal neurons. Pyramidal neurons expressed both CB and CR in layers II, III, V, and IV. However, there was a population of large pyramidal-shaped CR-ir neurons in layers V and VI that were significantly larger than those observed in the same layers of CB preparations. In addition, we identified three main types of non-pyramidal CBPs-ir neurons: spheroidal, polygonal, and fusiform. Non-pyramidal neurons immunoreactive for CR, CB, and PV are similar, except for fusiform neurons containing CR, which were usually smaller than those immunoreactive for CB and, especially, PV. The presence of CR-ir neurons strongly suggests a GABAergic neuronal population (Glezer et al., 1992). Their presence may also indicate that the absence of a distinct layer IV, as generally expressed in the primate and rodent neocortex, could be replaced here by a *diffuse band* of GABAergic/CR-ir neurons (Graïc et al., 2021), although this specific aspect required further investigation. Overall, our observations, combined with those concerning the laminar distribution, suggest that PV, CB, and CR are primarily localized in non-overlapping neuronal populations in the dolphin entorhinal cortex.

Our immunohistochemical observations are consistent with previous studies in rodents and primates (Tuñón et al., 1992; Schmidt et al., 1993; Seress et al., 1994; Wouterlood et al., 1995, 2000; Fujimaru and Kosaka, 1996; Miettinen et al., 1996, 1997; Mikkonen et al., 1997; Berger et al., 1999; Suzuki and Porteros, 2002; Grateron et al., 2003; Kobro-Flatmoen and Witter, 2019). As in terrestrial mammals, CB and PV are primarily expressed in neurons located in layers II and III, whereas the CR-ir neurons are distributed throughout the layers, and especially in layers V and VI. Studies in terrestrial mammals (Kobro-Flatmoen and Witter, 2019) show that layer I is devoid of PV-ir neurons, but in our study layer I of the dolphin entorhinal cortex contained PV-ir neurons.

In rodents and primates, the distribution of CBPs-ir neurons is highly dependent on the entorhinal subfield analyzed (Tuñón et al., 1992; Schmidt et al., 1993; Seress et al., 1994; Wouterlood et al., 1995, 2000; Fujimaru and Kosaka, 1996; Miettinen et al., 1996, 1997; Mikkonen et al., 1997; Berger et al., 1999; Suzuki and Porteros, 2002; Grateron et al., 2003; Kobro-Flatmoen and Witter, 2019). However, in the present study in the bottlenose dolphin, we noted some differences between the cytoarchitectonic organization of the LEA and MEA (see above), but the distribution of the immunoreactivity for the CR, CB and PV was similar in the two subdivisions of the periarchicortex.

Layers II and III of the entorhinal cortex provide the main cortical input to the hippocampal formation, while layers V and VI receive information from the hippocampal formation and transmit it to the neocortex and other brain structures (Witter and Amaral, 1991; Insausti et al., 1997; van Groen et al., 2003; Kerr et al., 2007; Witter, 2007, 2012; Insausti and Amaral, 2012; Cappaert Van Strien and Witter, 2015; Witter et al., 2017). Our results indicate that calcium-binding protein neurons in the dolphin entorhinal cortex are located in the interface between entorhinal input and output pathways. CB-ir pyramidal neurons in layers II and III may harbor output neurons that project through the perforant pathway to the dentate gyrus and CA1-3 regions of the hippocampus proper. Taken together, our data suggest that pyramidal neurons immunoreactive for the CR in layers V and VI could be projection neurons involved in signal flow between different regions of the hippocampal formation. Non-pyramidal PV-ir neurons, together with those immunoreactive for CB and CR, could act as local interneurons that directly or indirectly regulate the activity of projection cells. Contemplating the entorhinal cortex contextually, since the seminal work from Hafting and colleagues (Hafting et al., 2005) describing a topographical orientation map in the entorhinal cortex of rodents, the entorhinal cortex of cetaceans, a taxa that lives in the ocean with very few external landmarks, could prove to be a very interesting comparative neuroanatomical example. As mentioned in the introduction, dolphins do not possess an olfactory bulb and are indeed deprived of olfaction [but not necessarily of chemoreception; for reference see Cozzi et al. (2017)]. This lack of function may call into question the role of the entorhinal formation and lamina dissecans in cetaceans, as they are usually considered in terrestrial mammals. A working hypothesis is that the entorhinal formation may be the target of projections originating from other sensory areas, possibly related to the establishment of connections to and between the amygdalae. The topography of the sensory areas responsible for echolocation, which map and interpret sound emission and perception into distance and shape, is currently uncertain at best. A recent study (Gerussi et al., 2023) found that the dolphin prefrontal cortex occupies the cranio-lateral, ectolateral and opercular gyri, with projections involving lateral and ventral parts of the forebrain, hence close to the area presently discussed. A specific study using tractography may help the issue and evaluate the contribution of the entorhinal area to the rest of the cerebral network in dolphins.

## Data availability statement

The raw data supporting the conclusions of this article will be made available by the authors, without undue reservation.

## Ethics statement

Ethical approval was not required for the study involving humans in accordance with the local legislation and institutional requirements. Written informed consent to participate in this study was not required from the participants or the participants' legal guardians/next of kin in accordance with the national legislation and the institutional requirements. Ethical approval was not required for the study involving animals in accordance with the local legislation and institutional requirements because Dolphin brains were extracted during routine necropsy performed at the Department of Comparative Biomedicine and Food Science (BCA) of the University of Padova (Italy) on specimens. The brains were consequently fixed in phosphate buffered paraformaldehyde (4%), cut in coronal slices (about 1.5 cm × 2.5 cm) and stored in the Mediterranean marine mammal tissue bank (MMMTB, http://www.marinemammals.eu), located in BCA. The MMMTB is a CITES recognized (IT020) research center of the University of Padova, sponsored by and collaborating with the Italian Ministry of the Environment. MMMTB collects and stores samples from wild or captive marine mammals whose samples or whole carcasses are delivered to BCA for post-mortem diagnostics.

## Author contributions

J-MG: Conceptualization, Data curation, Investigation, Methodology, Supervision, Writing – original draft, Writing – review & editing. AG: Conceptualization, Data curation, Data curation, Supervision, Validation, Writing – review & editing. SS: Data curation, Supervision, Validation, Writing – review & editing. CT: Data curation, Investigation, Methodology, Software, Writing – review & editing. GS: Data curation, Investigation, Methodology, Software, Writing – review & editing. BC: Conceptualization, Data curation, Formal analysis, Resources, Supervision, Writing – review & editing. CB: Conceptualization, Data curation, Investigation, Resources, Supervision, Validation, Writing – original draft, Writing – review & editing.
